# Cardiac electrical abnormalities in childhood acute lymphoblastic leukemia survivors: a systematic review

**DOI:** 10.1186/s40959-023-00188-9

**Published:** 2023-11-11

**Authors:** Émilie Bertrand, Maxime Caru, Audrey Harvey, Philippe Dodin, Vincent Jacquemet, Daniel Curnier

**Affiliations:** 1 Laboratory of Pathophysiology of EXercise (LPEX), School of Kinesiology and Physical Activity Sciences, Faculty of Medicine, University of Montreal, 2100, Boulevard Édouard Montpetit, Montreal, QC H3C 3J7 Canada; 2 Sainte-Justine University Health Center, Research Center, Montreal, Canada; 3 Department of Pediatrics, Division of Hematology and Oncology, Penn State Health Children’s Hospital & Department of Public Health Sciences, Penn State College of Medicine, Hershey, USA; 4 Department of Pharmacology and Physiology, Faculty of Medicine, University of Montreal, Montreal, Canada

**Keywords:** Acute lymphoblastic leukemia, Pediatric cancer survivorship, Electrocardiogram, Cardiotoxicity

## Abstract

**Purpose:**

The aim was to provide evidence about the prevalence, incidence, and risk factors of cardiac electrical abnormalities in childhood acute lymphoblastic leukemia (ALL) survivors.

**Methods:**

We included all original studies reporting the incidence and/or prevalence of cardiac electrical abnormalities and/or risk factors associated with cardiac electrical abnormalities in childhood ALL survivors (< 21 years old at the time of their initial cancer diagnosis) who were post-treatment. Searches of the databases PubMed, Ovid MEDLINE(R) and Epub Ahead of Print, In-Process, In-Data-Review & Other Non-Indexed Citations, Daily and Versions(R), Ovid All EBM Reviews, Ovid Embase, and ISI Web of Science were completed in May 2023. The risk of bias was assessed using the standard JBI critical appraisal checklists.

**Results:**

The 11 studies included in this review (*N* = 1,264 participants) evaluated various parameters, including different cardiac electrical abnormalities. Five studies reported heart rate abnormalities (0–68%), six reported repolarization disorders (0–30%), two reported depolarization disorders (0–1%), seven reported rhythm disturbances or abnormalities (0–100%), four reported conduction disorders (0–10%), and three reported unclassified abnormalities (1–38%). No risk factors were reported.

**Conclusions:**

Electrical heart problems have been observed in childhood ALL survivors after completion of treatment. Large prospective studies in childhood ALL survivors, clear definitions of cardiac electrical abnormalities, and comparison with a control group are warranted.

**Implications for cancer survivors:**

Cardiac electrical abnormalities induced by chemotherapy-related cardiotoxicity in the growing population of childhood ALL survivors need to be better characterized to ensure better long-term follow-up and improve overall survival rate.

**Supplementary Information:**

The online version contains supplementary material available at 10.1186/s40959-023-00188-9.

## Introduction

Acute lymphoblastic leukemia (ALL) is the most prevalent cancer in children [1]. In current anti-cancer treatments, most chemotherapy protocols are multidrug regimens associated with other treatments (e.g., radiotherapy and stem cell transplantation). Such combinations have been successful and have increased current survival rates (i.e., the 5-year relative survival rate is ~ 90%) [2]. Nevertheless, studies showed an unexpectedly high incidence of late myocardial damage in long-term survivors of childhood cancer [3–7].

A high rate of late cardiotoxicity and a high risk of clinical dysfunction in childhood ALL survivors [3, 8–12] is documented. These events are known to be related to the anthracycline cumulative doses [13, 14]. Late cardiotoxicity is defined as cardiovascular disease and cardiovascular morbidity and mortality, such as impaired left ventricular contractility, high-grade ectopy, late congestive heart failure, and sudden death [10, 15, 16]. Studies have also reported cardiac electrical problems many years after the end of anthracycline therapy, such as late development of subclinical cardiac dysfunction [17]. Thus, electrical heart problems can be caused by anti-cancer treatments and can be a precursor to more serious cardiac problems. Resulting manifestations appear after a prolonged asymptomatic period of one or more years before becoming detectable [5]. By that time, they have often already evolved into clinically significant diseases, such as cardiomyopathy or congestive heart failure.

In addition to chemotherapy treatment, there are several risk factors for late cardiotoxicity, such as female sex [18], young age at diagnosis [3, 19], cumulative cardiac radiation dose > 5 Gy [13], pre-existing cardiac risk factors [20], personal health habits [21, 22], and genetic factors [23–28]. Concurrent treatments also increase the risk of cardiotoxicity [29]. In fact, radiation therapy delivered to the thoracic region and chemotherapy together are more harmful to the heart than these treatments administered separately [29].

Treatment protocols involving anthracycline dosage limitation and less radiotherapy have been developed to minimize the likelihood of late cardiotoxicity [30, 31]. Moreover, published research highlights the importance of life-long follow-up of childhood ALL survivors to detect early signs of subclinical cardiac damage [32–36]. Thus, cardiac monitoring (e.g., ejection fraction measured by echocardiography) is used to enhance early detection of cardiac dysfunction in childhood ALL survivors with doses ≥ 250 mg/m^2^ [37]. The resting echocardiogram is the standard follow-up procedure used to detect changes in cardiac structure and function after treatment [37, 38]. However, it has been questioned whether these methods are sensitive enough to detect subclinical cardiac dysfunction, with no consensus on the most optimal screening tool [39, 40]. Considering that the incidence of negative cardiac effects in childhood cancer survivors is higher as post-treatment follow-up periods increase [3, 41], it is of interest to study this area. Moreover, a better understanding and characterization of chemotherapy-related cardiotoxicity in childhood ALL survivors are necessary to insure a better long-term follow-up to prevent the progression of potential heart diseases.

## Review aims and questions

The first aim of this review was to provide evidence of the prevalence and incidence of cardiac electrical abnormalities in childhood ALL survivors. The second aim was to evaluate which risk factors are associated with cardiac electrical abnormalities in childhood ALL survivors. We formulated the following question: In post-treatment childhood ALL survivors, what is the prevalence and incidence of cardiac electrical abnormalities? And which risk factors are associated with cardiac electrical abnormalities?

## Methods

A preliminary search of PROSPERO, MEDLINE, the Cochrane Database of Systematic Reviews, and *JBI Evidence Synthesis* was performed (date of consultation: 11–01-2021) and no current or in-progress systematic reviews on the topic were identified. This systematic review was conducted in accordance with the Preferred Reporting Items for Systematic Reviews and the Meta-Analyses (PRISMA) statement (2020) [42]. This review was also conducted in accordance with an a priori protocol registered through Prospero (CRD42022326019) [43].

### Inclusion criteria

#### Participants

We included all original studies reporting the incidence and/or prevalence of cardiac electrical abnormalities in post-treatment childhood ALL survivors who were < 21 years old at the time of their initial cancer diagnosis. There was no restriction regarding the type of cancer treatments.

#### Outcomes

Participants with an electrocardiographic evaluation (e.g., standard 12-lead electrocardiogram (ECG) and 24-h ambulatory ECG) performed at any time after their last exposure to cancer treatment were included. Cardiac electrical abnormality is defined as alterations in multiple electrogenic transport processes within the cardiac myocyte [44]. It encompasses electrophysiological changes, heart rate impairment, and ECG abnormalities including, but not limited to, prolonged QT interval, prolonged QT dispersion, increase P wave duration, increase P wave dispersion, fragmented QRS, and arrhythmias.

#### Types of studies

Conference abstracts, case reports, short communications, systematic reviews, meta-analyses, theses, letters to the editor, and protocol papers were excluded. Animal studies were also excluded from the search. We included observational studies, cross-sectional studies, retrospective studies, prospective cohort studies, case–control studies, randomized controlled trials, nonrandomized controlled trials, and uncontrolled interventions (i.e., pre- and post-tests without controls).

### Search strategy

The search strategy aimed to locate published original articles. The search strategy, including all identified keywords and index terms, was adapted for each included information source. Searches of the databases PubMed, Ovid MEDLINE(R) and Epub Ahead of Print, In-Process, In-Data-Review & Other Non-Indexed Citations, Daily and Versions(R), Ovid All EBM Reviews, Ovid Embase, and ISI Web of Science were completed by a librarian (PD) of Sainte-Justine University Health Center with special training and skills in literature searches in May 2023 (Supplementary Tables S1, S2, S3, S4, and S5). The search terms for the inclusion criteria were a combination of database specific MeSH terms and keywords (Table 1). The references were manually scanned in all identified articles for additional studies. No limits were applied to publication dates. Only articles written in French, English or Spanish were included. Duplicates were removed in EndNote (Clarivate Analytics, PA, USA) by the librarian (PD).

**Table 1:** Search terms for the inclusion criteria

#1	Leukemia	Leukemia[mh] OR Hematologic Neoplasms[mh:noexp] OR Leukemi*[tiab] OR Leucocythaemi*[tiab] OR Leucocythemi*[tiab] OR Hematologic Neoplasm*[tiab] OR Haematologic Neoplasm*[tiab] OR Hematopoietic Neoplasm*[tiab] OR Haematopoietic Neoplasm*[tiab] OR Hematological Neoplasm*[tiab] OR Haematological Neoplasm*[tiab] OR Hematologic Cancer*[tiab] OR Haematologic Cancer*[tiab] OR Hematopoietic Cancer*[tiab] OR Haematopoietic Cancer*[tiab] OR Hematological Cancer*[tiab] OR Haematological Cancer*[tiab] OR Hematologic Malignanc*[tiab] OR Haematologic Malignanc*[tiab] OR Hematopoietic Malignanc*[tiab] OR Haematopoietic Malignanc*[tiab] OR Hematological Malignanc*[tiab] OR Haematological Malignanc*[tiab] OR Hematologic Tumor*[tiab] OR Haematologic Tumor*[tiab] OR Hematopoietic Tumor*[tiab] OR Haematopoietic Tumor*[tiab] OR Hematological Tumor*[tiab] OR Haematological Tumor*[tiab] OR Hematologic Tumour*[tiab] OR Haematologic Tumour*[tiab] OR Hematopoietic Tumour*[tiab] OR Haematopoietic Tumour*[tiab] OR Hematological Tumour*[tiab] OR Haematological Tumour*[tiab] OR blood cancer*[tiab] OR blood neoplasm*[tiab] OR blood tumor*[tiab] OR blood tumour*[tiab] OR blood malignan*[tiab]
#2	Pediatric	Infant[MH] OR Child[MH] OR Adolescent[MH] OR Intensive Care Units, Pediatric[MH] OR Hospitals, Pediatric[MH] OR Pediatrics[MH] OR Pediatricians[MH] OR Child, Hospitalized[MH] OR Adolescent, Hospitalized[MH] OR newborn*[tiab] OR new born*[tiab] OR babie*[tiab] OR baby*[tiab] OR infant*[tiab] OR infancy[tiab] OR toddler*[tiab] OR preschool*[tiab] OR pre school*[tiab] OR child*[tiab] OR kid[tiab] OR kid'[tiab] OR kids[tiab] OR kid's[tiab] OR boy[tiab] OR boy'[tiab] OR boys[tiab] OR boy's[tiab] OR girl[tiab] OR girl'[tiab] OR girls[tiab] OR girl's[tiab] OR schoolchild*[tiab] OR juvenil*[tiab] OR preadolescen*[tiab] OR youth*[tiab] OR adolescen*[tiab] OR teen*[tiab] OR puber*[tiab] OR high school*[tiab] OR highschool*[tiab] OR secondary school*[tiab] OR paediatric*[tiab] OR pediatric*[tiab] OR PICU*[tiab] OR neonat*[tiab] OR neo nat*[tiab]
#3	Pediatric cancer	(Neoplasms[mh:noexp] AND child[mh]) OR Childhood cancer*[tiab] OR Pediatric cancer*[tiab] OR Paediatric cancer*[tiab] OR Childhood neoplasm*[tiab] OR Pediatric neoplasm*[tiab] OR Paediatric neoplasm*[tiab] OR Childhood malignanc*[tiab] OR Pediatric malignanc*[tiab] OR Paediatric malignanc*[tiab] OR Childhood tumor*[tiab] OR Pediatric tumor*[tiab] OR Paediatric tumor*[tiab] OR Childhood tumour*[tiab] OR Pediatric tumour*[tiab] OR Paediatric tumour*[tiab]
#4	Heart	Cardiovascular Diseases[mh] OR Heart[mh] OR heart[tiab] OR cardia*[tiab] OR cardio*[tiab] OR arrythm*[tiab] OR arrhythm*[tiab] OR dysrhythm*[tiab] OR myocard*[tiab] OR Pericardi*[tiab] OR Ventric*[tiab] OR endocard*[tiab]? OR tachycardi*[tiab] OR Tachyarr*[tiab]
#5	Cardiac remodeling	Cardiomegaly[mh] OR Ventricular remodeling[mh] OR Remodel*[tiab] OR repolari*[tiab] OR ((interval[tiab] OR QT[tiab] OR QTc[tiab]) AND (prolong*[tiab] OR dispers*[tiab] OR shorten*[tiab] OR longer[tiab])) OR ((electrocardio*[tiab] OR echocardio*[tiab] OR electro-cardio*[tiab] OR echo-cardio*[tiab] OR ECG[tiab] OR ECGs[tiab] OR EKG[tiab]) AND (abnormal*[tiab] OR anormal*[tiab] OR anomal*[tiab])) OR wall thickness[tiab] OR Decompensat*[tiab] OR Hypertroph*[tiab] OR Dilation[tiab] OR Dilatation[tiab] OR cardiomegal*[tiab] OR enlarge*[tiab] OR expansion[tiab]

### Study selection

All records identified from the search strategy were collated and uploaded into the Covidence systematic review software (Veritas Health Innovation, Melbourne, Australia). Duplicate articles were removed by the bibliographic software and manually scanned. Two independent reviewers (EB, MC) screened the titles and abstracts to remove additional duplicates and to confirm reliability with the eligibility criteria. When the title and the abstract were considered relevant, the full text was obtained. Full text papers were analyzed to confirm their eligibility in accordance with the inclusion criteria by the same two authors. Full-text studies that did not meet the inclusion criteria were excluded, and the reasons for their exclusion are provided in Supplementary Table S6. In case of disagreement, articles were re-examined and discussed. Authors of the included papers were contacted to request missing or additional data for clarification, where required.

### Assessment of risk of bias

Eligible studies were critically appraised by the lead author (EB) and revised by a second reviewer (MC) for methodological quality using the standard JBI critical appraisal checklists specific to the study design of all included studies [45, 46]. Any disagreements that arose between the reviewers were resolved through discussion.

### Data extraction

To maintain the integrity of the data, the lead author (EB) extracted data from each eligible article, while another author (MC) verified the extracted data. Any disagreements that arose between the reviewers were resolved through discussion or with the help of a third reviewer. However, no discrepancies and/or disagreements occurred in the data extraction. Authors of the papers were contacted to request missing or additional data, where required (Supplementary Table S7).

The extracted data included specific details about the authors and the publication year of the article, study design, study aims, childhood ALL survivors’ characteristics (number of participants, sex, geographical region, socio-economic status, ethnicity, comorbidities, age at cancer diagnosis, age at cardiac evaluation, time between end of cancer treatment and follow-up), details of cancer history (cancer treatment received and the absolute cumulative dosage, length of follow-up, cardioprotective treatment, e.g. dexrazoxane), authors' definition of cardiac electrical abnormalities, method of detection of cardiac electrical abnormalities, method of follow-up, risk factors (as defined by the authors of the included studies), effect sizes, and incidence and/or prevalence of cardiac electrical abnormalities.

## Results

### Study search and study characteristics

**Fig. 1:**
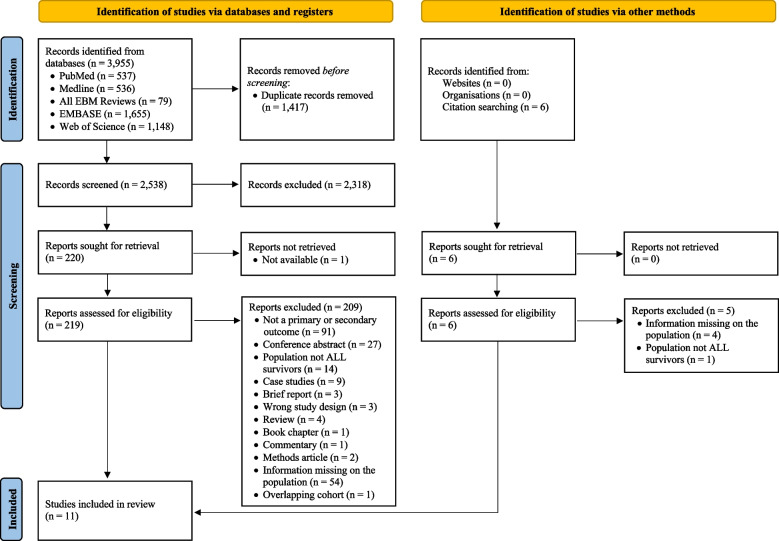
PRISMA flow diagram

The literature search resulted in 3,955 articles of which 48 articles were retrieved in full text (Fig. 1). Among them, 37 studies were not included in this review because they included different types of childhood cancer, and we were not able to obtain missing information about the specific characteristics of childhood ALL survivors from the corresponding authors (Supplementary Table S7). One study was excluded because of overlapping cohorts [47] and the most recent study was chosen [48].

Table 2 provides detailed information about the 11 included studies. Among them, 9 studies were specific to only childhood ALL and 2 studies included other types of cancer (ALL, acute promyelocytic leukemia, embryonal rhabdomyosarcoma, Ewing’s sarcoma, Hodgkin’s disease, osteogenic sarcoma, acute myeloblastic leukemia, neuroblastoma, and aplastic anemia) [48, 49].

**Table 2:** Characteristics of included studies assessing prevalence and incidence of cardiac electrical abnormalities

Authors	Study design	Study aims	Demographics and baseline characteristics	Cancer characteristics
Bertrand et al., 2021 [50]	Cross-sectional study	To examine heart rate response during a maximal cardiopulmonary exercise test	**Participants** (*N* = 216) **Eligible for review** *N* = 216 (51.4% males) **Age at cardiac evaluation (years)** mean ± SD: 22.0 ± 6.4 median (range): 22.0 (8.0—40.0) **Geographical region:** Canada **Socio-economic status:** Not reported* **Ethnicity:** French-Canadian descent **Comorbidities:** Not reported*	**Period of diagnosis:** 1987—2010 **Age at cancer diagnosis (years)** mean ± SD: 6.4 ± 4.7 median (range): 4.0 (0.9—18.0) **Time since the end of cancer treatment:** minimum 5 years **Length of follow-up:** N/A **Cancer treatment and absolute cumulative dosage** Anthracycline chemotherapy (doxorubicin) (*N* = 216) mean ± SD: 85.4 ± 121.7 mg/m^2^ median (range): 230.9 (43.6—472.9) mg/m^2^ Cranial radiotherapy (*N* = 123) no dosage specified **Cardioprotective treatment:** Dexrazoxane (*N* = 65)
Brouwer et al., 2007 [51]	Cross-sectional study	To re-assesses cardiac status at a very long follow-up of median 22 years post treatment in a subset of the earlier studied survivors	**Participants** (*N* = 23) **Eligible for review** *N*** = **23 (39.1% males) **Age at cardiac evaluation (years)** median (range): 29.0 (24.0—39.0) **Geographical region:** Netherlands **Socio-economic status:** Not reported* **Ethnicity:** Not reported **Comorbidities:** Not reported*	**Period of diagnosis:** Not reported **Age at cancer diagnosis (years)** median (range): 5.0 (2.0—14.0) **Time since the end of cancer treatment (years)** median (range): 22.0 (19.5—24.5) **Length of follow-up:** N/A **Cancer treatment and absolute cumulative dosage** Induction treatment (6 weeks) Anthracycline (daunorubicin) (*N* = 13): 100 mg/m^2^/week Vincristine: 6 × 2 mg/m^2^/week Prednisone: 28 × 40 mg/m^2^/day L-Asparaginase: 14 × 200 E/kg/day (weeks 4–6) Central nervous system prophylaxis (2.5–3 weeks) Cranial radiotherapy (*N* = 23): median (range): 25 (18—25) Gy Methotrexate: 12.5 mg/m^2^ (maximum 15 mg/m^2^) Prednisone: 5 × 12.5 mg/m^2^ Maintenance and consolidation phase (until 2 years from start of treatment) 6-mercaptopurine: 50 mg/m^2^/day for 5 weeks Alternating with 2 weeks: Methotrexate: 30 mg/m^2^/week OR Vincristine: 2 mg/m^2^/week Prednisone: 40 mg/m^2^/day **Cardioprotective treatment:** Not reported
Halazun et al., 1974 [52]	Randomized controlled trial	To describe the evidence to support the occurrence of cardiotoxicity from daunorubicin	**Participants** (*N* = 172) **Eligible for review** *N* = 172 (58.1% males) **Age at cardiac evaluation:** Not reported **Geographical region:** France, USA **Socio-economic status:** Not reported* **Ethnicity:** Not reported* **Comorbidities:** Not reported*	**Period of diagnosis:** 1968–1971 **Age at cancer diagnosis:** Not reported **Time since the end of cancer treatment:** median: 79 days range: 14–280 days **Length of follow-up:** Not reported **Cancer treatment and absolute cumulative dosage** Anthracycline chemotherapy (daunorubicin) mean: 780 mg/m^2^ range: 360–1260 mg/m^2^ Methotrexate no dosage specified L-Asparaginase no dosage specified Vincristine no dosage specified Prednisone no dosage specified **Cardioprotective treatment:** Not reported
Hau Eva et al., 2019 [53]	Case control study	To compare the risk of cardiovascular disease reported by acute lymphoblastic leukemia survivors to that of their siblings, and changes in risk based on the calendar period of diagnosis. It also examines treatment-related risk factors for cardiovascular disease	**Participants** (*N* = 511) **Eligible for review** *N* = 511 (50.5% males) **Age at cardiac evaluation (years)** range 16—20 (*N* = 135) range 21—30 (*N* = 251) range 31—40 (*N* = 107) range 41 or more (*N* = 18) **Geographical region:** Swiss **Socio-economic status:** Not reported* **Ethnicity:** Not reported **Comorbidities:** Not reported*	**Period of diagnosis:** 1976—2005 **Age at cancer diagnosis (years)** (range): (0—19) **Time since the end of cancer treatment:** minimum 5 years **Length of follow-up:** N/A **Cancer treatment and absolute cumulative dosage** Anthracycline (*N* = 315): no dosage specified Radiotherapy (*N* = 151): no dosage specified Chest radiotherapy (*N* = 23): no dosage specified Hematopoietic stem cell transplantation (*N* = 23) **Cardioprotective treatment:** Not reported
Lipshultz et al., 1991 [3]	Cross-sectional study	To evaluate cardiac status in children 1 to 15 years after the successful treatment of acute lymphoblastic leukemia with chemotherapeutic regimens that included doxorubicin	**Participants** (*N*** = **115) **Eligible for review** *N* = 115 (50.4% males) **Age at cardiac evaluation (years)** mean: 13.6 median (range): 12.6 (3.9—31.7) **Geographical region:** USA **Socio-economic status:** Not reported* **Ethnicity:** Not reported **Comorbidities:** Not reported*	**Period of diagnosis:** 1972—1987 **Age at cancer diagnosis** mean: 6.3 years median (range): 4.8 years (7 months—19.1 years) **Time since the end of cancer treatment (years)** minimum one median (range): 6.4 (1.0—15.0) **Length of follow-up:** N/A **Cancer treatment and absolute cumulative dosage** Anthracycline (doxorubicin) (*N* = 18): 45 mg/m^2^ Anthracycline (doxorubicin) (*N* = 97): median (range): 360 (228–550) mg/m^2^ Vincristine: no dosage specified Methotrexate: no dosage specified Prednisone: no dosage specified Mercaptopurine: no dosage specified l-asparaginase: no dosage specified **Cardioprotective treatment:** Not reported
Pihkala et al., 1994 [49]	Cohort study	To evaluate the long-term effects of bone marrow transplant on myocardial function in children	**Participants** (*N* = 30) **Eligible for review** *N* = 9 (44.4% males) **Age at cardiac evaluation:** Not reported **Geographical region:** Finland **Socio-economic status:** Not reported* **Ethnicity:** Not reported **Comorbidities:** Not reported*	**Period of diagnosis:** Not reported **Age at cancer diagnosis:** Not reported **Time since the end of cancer treatment (years)** mean ± SD: 5.5 ± 3.3 median (range): 5.9 (0.5–9.6) **Length of follow-up:** N/A **Cancer treatment and absolute cumulative dosage** Anthracycline chemotherapy (*N* = 9) mean ± SD: 174.4 ± 87.6 mg/m^2^ median (range): 140 (120—400) mg/m^2^ Total body irradiation (*N* = 9) mean ± SD: 1110.0 ± 101.7 cGy median (range): 1200 (970—1200) cGy Cyclophosphamide (*N* = 6): no dosage specified Cytosine arabinoside (*N* = 3): no dosage specified Allogenic bone marrow transplantation (*N* = 9) **Cardioprotective treatment:** Not reported
Velensek Prestor et al., 2000 [17]	Cross-sectional study	To analyze the influence of risk factors to anthracycline cardiotoxicity and to define the most suitable method of late cardiac toxicity detection	**Participants** (*N* = 46) **Eligible for review** *N* = 46 (37.8% males) **Age at cardiac evaluation (years)** mean: 23.0 range: 18.0—33.0 **Geographical region:** Slovenia **Socio-economic status:** Not reported* **Ethnicity:** Not reported **Comorbidities:** Not reported*	**Period of diagnosis:** 1968—1992 **Age at cancer diagnosis (years)** mean: 7.0 range: 2.0—17.0 **Time since the end of cancer treatment:** minimum 5 years mean: 16 range: 5—23 **Length of follow-up:** N/A **Cancer treatment and absolute cumulative dosage** Anthracycline chemotherapy (*N* = 34) mean: 203.0 mg/m^2^ range: 50.0—540.0 Cranial radiotherapy (*N* = 46) range: 12—24 Gy AA-cyclophosphamide (*N* = 9): 3 × 1000 mg/m^2^ AA-cyclophosphamide (*N* = 1): 2 × 600 mg/m^2^ AA-cyclophosphamide (*N* = 3): 8400 mg/m^2^ Vincristine: no dosage specified Methotrexate: no dosage specified Prednisone: no dosage specified Mercaptopurine: no dosage specified l-asparaginase: no dosage specified Cytarabine: no dosage specified Thioguanine: no dosage specified **Cardioprotective treatment:** Not reported
Rammeloo et al., 2000 [54]	Randomized control trial	To investigate late cardiotoxicity in childhood acute lymphoblastic leukemia survivors after induction treatment with or without daunorubicin	**Participants** (*N* = 90) **Eligible for review** *N* = 90 (44.4% males) **Age at cardiac evaluation (year)** Group A: median (range): 18.6 (14.7—31.3) Group B: median (range): 20.1 (14.8—30.0) **Geographical region:** Netherlands **Socio-economic status:** Not reported* **Ethnicity:** Not reported **Comorbidities:** Not reported*	**Period of diagnosis:** 1979—1984 **Age at cancer diagnosis (years)** median (range): 4.5 (1.2—14.9) **Time since the end of cancer treatment (years)** median (range): 14.8 (11.4—17.8) **Length of follow-up:** N/A **Cancer treatment and absolute cumulative dosage** Vincristine (*N* = 90): no dosage specified Prednisone (*N* = 90): no dosage specified Asparaginase (*N* = 90): no dosage specified Anthracycline (daunorubicin) (*N* = 50, group B): 100 mg/m^2^ **Cardioprotective treatment:** Not reported
Shimomura et al., 2011 [55]	Cross-sectional study	To evaluate Pirarubicin-induced late cardiotoxicity for asymptomatic children who received THP therapy in three consecutive JCCLSG studies (ALL911/ALL941/ALL2000)	**Participants** (*N* = 61) **Eligible for review** *N* = 61 (49.2% males) **Age at cardiac evaluation (years)** mean ± SD: 14.7 ± 3.5 median (range): 14.7 (7.6—25.7) **Geographical region: Japan** **Socio-economic status:** Not reported* **Ethnicity:** Not reported **Comorbidities:** Not reported*	**Period of diagnosis:** 1991–2003 **Age at cancer diagnosis (years)** mean ± SD: 5.7 ± 3.5 **Time since the end of cancer treatment (years)** mean ± SD: 7.2 ± 2.8 median (range): 8.1 (1.7—12.5) **Length of follow-up:** N/A **Cancer treatment and absolute cumulative dosage** Anthracycline (Pirarubicin (tetrahydropyranyl-adriamycin)) mean ± SD: 299 ± 192 mg/m^2^ median (range): 180 (120—740) mg/m^2^ Anthracycline chemotherapy (doxorubicin and Pirarubicin) mean ± SD: 346 ± 206 mg/m^2^ median (range): 207 (135—812) mg/m **Cardioprotective treatment:** Not reported
Steinherz et al., 1995 [48]	Cross-sectional study	To describe the clinical course, evolution of cardiac findings, and treatment of our symptomatic patients	**Participants** (*N* = 15) **Eligible for review** *N* = 2 (50% males) **Age at cardiac evaluation:** Not reported **Geographical region:** USA **Socio-economic status:** Not reported* **Ethnicity:** Not reported **Comorbidities:** Not reported*	**Period of diagnosis:** Not reported **Age at cancer diagnosis:** Not reported **Time since the end of cancer treatment:** 12 and 19 years **Length of follow-up:** N/A **Cancer treatment and absolute cumulative dosage** Anthracycline (daunorubicin) (*N* = 1): 795 mg/m^2^ Anthracycline (doxorubicin) (*N* = 1): 350 mg/m2 **Cardioprotective treatment:** Not reported
Turner-Gomes et al., 1996 [56]	Cross-sectional study	To assess cardiopulmonary status and exercise capacity after successful treatment	**Participants** (*N* = 19) **Eligible for review** *N* = 19 (47.4% males) **Age at cardiac evaluation (years)** mean ± SD: 13.0 ± 3.5 median (range): 12.3 (7.7—23.8) **Geographical region:** USA **Socio-economic status:** Not reported* **Ethnicity:** Not reported **Comorbidities:** Not reported*	**Period of diagnosis:** 1984—1990 **Age at cancer diagnosis (years)** mean ± SD: 6.1 ± 4.3 median (range): 4.6 (1.5—17.7) **Time since the end of cancer treatment (years)** Minimum one mean ± SD: 4.6 ± 1.5 median (range): 4.4. (1.1—7.1) **Length of follow-up:** N/A **Cancer treatment and absolute cumulative dosage** Anthracycline (doxorubicin): (*N* = 7 standard risk) mean ± SD: 50 ± 21 mg/m^2^ Anthracycline (doxorubicin): (*N* = 12 high risk/very high risk) mean ± SD: 349 ± 16 mg/m^2^ **Cardioprotective treatment:** Not reported

### Patient characteristics

All studies included participants who were diagnosed with childhood ALL. A total of 1,264 participants were eligible for the review. No studies reported socio-economic status and comorbidities of participants. Age at cancer diagnosis was extracted from eight studies, which ranged from 0 to 19 years old [3, 17, 50, 51, 53–56]. The period of cancer diagnosis ranged from 1968 to 2010 and was specified in eight studies [3, 17, 50, 52–56]. The time since the end of cancer treatment was reported in all studies as the median (ranging from 4.4 to 22.0 years), as the mean (ranging from 14 days to 15.5 years), or as the minimum (the minimum time since the end of treatment of included patients was 14 days). The age at cardiac evaluation was provided as the median or mean, the median varying between 12.3 and 29.0 years of age, and the mean varying between 13.0 and 23.0 years of age. The age of childhood ALL survivors eligible for review ranged from 3.9 to > 41.0 years and was not reported in three studies [48, 49, 52].

### Treatments characteristics

Childhood ALL survivors received different combinations of cardiotoxic treatments (Table 2). Detailed information on actual received combinations were not always provided by the included studies. All studies included anthracyclines. Doses were reported as provided by the included studies, but the type of anthracycline used was not always specified. The most frequently used treatments were doxorubicin and daunorubicin. The actual received cumulative anthracycline doses were reported in 10 studies and were provided as the mean, median, or proportions/range [3, 17, 48–52, 54–56]. The cumulative anthracycline doses ranged from 0.0 to 1260.0 mg/m^2^ (median ranging from 140.0 to 572.5 mg/m^2^ and mean ranging from 50.0 to 780.0 mg/m^2^).

Radiotherapy doses were reported in three [17, 49, 51] out of five [17, 49–51, 53] studies that included childhood ALL survivors treated with radiotherapy, and ranged from 9.7 to 25.0 Gy. Three studies reported the use of cranial radiotherapy [17, 50, 51], and one reported chest radiotherapy [53]. In one study, they specified that no patients underwent mediastinal radiation [3]. In the other four studies, it was not specified if childhood ALL survivors received radiotherapy.

Studies did not always specify all treatments. Among the other drugs received, there were vincristine, prednisone, asparaginase, cytarabine, thioguanine, cyclophosphamide, cytosine arabinoside, methotrexate, and mercaptopurine. In three studies, some participants also received stem cell transplantation [17, 49, 53]. One study reported the usage of a cardioprotective treatment (i.e., dexrazoxane) [50].

### Method of cardiac electrical abnormalities assessment

The included studies used different methods of cardiac electrical abnormalities detection, as presented in Table 3. Seven studies used a 12-lead ECG [17, 48, 49, 51, 52, 54, 55]. Four studies used a 12-lead ECG during an exercise test [3, 17, 50, 56]. Five studies used a 24-h ambulatory ECG [3, 48, 51, 54, 55]. One study used a questionnaire [53].

**Table 3:** Prevalence of cardiac electrical abnormalities

Authors	Assessment	Prevalence
Bertrand et al., 2021[50]	• 12-lead ECG during a maximal exercise test: heart rate	*N* = 147 (68.1%) (49.7% males) did not achieve their predicted maximal heart rate Maximal heart rate 97.3 ± 5.6% predicted
Brouwer et al., 2007 [51]	• 12-lead ECG: rhythm and conduction disturbances (Flattened T-waves, pathological Q-waves or a prolonged QTc. Ventricular arrhythmias were classified according to the Lown’s criteria. Lown 4 or higher was considered abnormal) • 24-h ambulatory ECG: rhythm and conduction disturbances (Flattened T-waves, pathological Q-waves or a prolonged QTc. Ventricular arrhythmias were classified according to the Lown’s criteria. Lown 4 or higher was considered abnormal)	12-lead ECG: - Flattened T-waves *N* = 7 (30%) - Pathological Q-waves *N* = 0 (0%) - Prolonged QTc *N* = 0 (0%) 24-h ambulatory ECG: - Sinus rhythm *N* = 23 (100%) - Normal atrioventricular conduction *N* = 23 (100%) - Sporadic (less than 100/24 h) premature ventricular contractions (Lown 1) *N* = 1 (4.3%) - Ventricular couplets (Lown 4) *N* = 2 (8.7%)
Halazun, 1974 [52]	• ECG	ECG abnormalities: *N* = 17 (9.9%) (males *N* = 10) Age (years) mean: 6.25 median (range): 5.00 (2.75–18.00) Low voltage T *N* = 15 Low voltage QRS *N* = 14 Abnormal T axis *N* = 11 Left atrial enlargement *N* = 10 Abnormal QRS axis *N* = 9 Bi-atrial enlargement *N* = 7 Abnormal Q *N* = 2 ST changes *N* = 2
Hau Eva et al., 2019 [53]	• Questionnaire: arrhythmia	Arrhythmia: - *N* = 27 (5.3%) - Missing values 1.1% Controls *N* = 21 3.0% (missing values 4.7%) OR: 1.8 95% CI: 1.0–3.5 *p*-value: 0.065
Lipshultz et al., 1991 [3]	• 24-h ambulatory ECG Holter (*N* = 89): abnormalities of heart rate and rhythm • 12-lead ECG during a maximal exercise test (*N* = 96): abnormalities of heart rate and rhythm	Holter: - Ventricular tachycardia *N* = 4 Exercise Test: - Excess tachycardia *N* = 8 - Atrial ectopy *N* = 3 - Ventricular ectopy *N* = 12 (Lown grade 1 through 4A) (10%) - Abnormal ST-segment and T-wave changes *N* = 9
Pihkala et al., 1994 [49]	• 12-lead ECG: Total QRS voltage (pre/follow-up) • Number of evaluations: 3 (pre diagnosis, 1–4 months after bone marrow transplantation and at follow-up)	- Change of QRS voltage from diagnosis to follow-up (%) significantly decrease (> 15%) *N* = 3 Cyclophosphamide + total body irradiation *N* = 2 Cytosine arabinoside + total body irradiation *N* = 1 - ST change (*N* = 0)
Velensek Prestor et al., 2000 [17]	• 12-lead ECG: rhythm and conduction disturbances • 12-lead ECG during a submaximal exercise test (*N* = 44): rhythm and conduction disturbances	12-lead ECG at rest and/or exercise: - Left ventricular hypertrophy: *N* = 2 12-lead ECG: - ECG changes: *N* = 13 - Nonspecific ST-T changes: *N* = 3 - QTc prolongation ≥ 0.43 s: *N* = 7 - QTc prolongation ≥ 0.45 s: *N* = 2 - Right bundle branch block: *N* = 1 - Supraventricular tachycardia: *N* = 2 - Sinus bradycardia: *N* = 4 12-lead ECG during exercise: - ECG changes: *N* = 13 - QTc prolongation: *N* = 2 - Ventricular premature complexes: *N* = 3 - Depression of ST interval: *N* = 10
Rammeloo et al., 2000 [54]	• 12-lead ECG: abnormalities (decreased QRS voltage, prolongation of the QTc interval Bazett, T wave inversion, ST-T abnormalities, and supraventricular and ventricular arrhythmias) • 24-h ambulatory ECG: rate, basal rhythm, atrioventricular conduction, and supraventricular and ventricular arrhythmias	12-lead ECG: - QTc interval > 0.44 s *N* = 0 - Group A: *N* = 1 prolonged QRS interval duration resulting from Wolff-Parkinson-White syndrome - Group B: *N* = 1 flattened T-waves in the chest leads. *N* = 1 low QRS voltage 24-h ambulatory ECG (*N* = 89; Group A: *N* = 40 and Group B: *N* = 49): - Second-degree AV block type I during sleep A: *N* = 0, B: *N* = 3 - Basal sinus rhythm A: *N* = 40, B: *N* = 46 - Premature atrial contractions > 100/24 h A: 2.5%, B: 6% - Premature ventricular contractions > 50/24 h A: 6%, B: 2% - Supraventricular or ventricular tachycardia *N* = 0
Shimomura et al., 2011 [55]	• ECG: abnormal ECG response was defined as a horizontal or downsloping ST segment depression of 0.10 mV (1 mm) for 80 ms • Holter ambulatory ECG • Number of evaluations: before, immediately following, and 1 min after exercise	ECG: - ECG normal (rest) *N* = 61 - ST elevation (after exercise) *N* = 1 (1.6%) Holter: - Arrhythmia *N* = 2/59 (3.3%) (supra-ventricular premature contraction)
Steinherz et al., 1995 [48]	• Medical records ○ ECG ○ 24-h taped electrocardiography	Ventricular ectopy *N* = 1 (50%) Ventricular dysrhythmia *N* = 2 (100%) Tachycardia *N* = 1 (50%) Ventricular premature contractions *N* = 1 (50%)
Turner-Gomes et al., 1996 [56]	Evaluation of heart rate during a maximal exercise test (no specification)	Maximal heart rate 97.4 ± 12.3% predicted No difference heart rate responses to maximal exercise in the high risk/very high risk (96.5, 15.1% predicted) versus standard risk group (98.7 ± 8.2% predicted) Normal limits of HRmax *N* = 19

*ECG* electrocardiogram

### Prevalence of cardiac electrical abnormalities

Information on the prevalence of cardiac electrical abnormalities is provided in Tables 3 and 4. Different cardiac electrical abnormalities were identified, which could be categorized as heart rate abnormalities (maximal heart rate, tachycardia, bradycardia), repolarization disorders (QT and QTc prolongation, abnormal ST segments and T-wave changes), depolarization disorders (pathologic Q-waves), rhythm disturbances or abnormalities (sinus rhythm, arrhythmias, atrial ectopy, ventricular arrhythmias [ventricular premature contractions, ventricular tachycardia, ventricular ectopy], premature atrial contractions, prolonged QRS interval), conduction disorders (abnormal atrioventricular [AV] contraction, second-degree AV block, right bundle branch block, atrial enlargement [abnormal P waves]), and unclassified disorders (low QRS voltage, abnormal QRS axis, low T voltage, abnormal T axis).

**Table 4:** Total prevalence of cardiac electrical abnormalities

Abnormalities	Bertrand et al. (2021) [50]	Brouwer et al. (2007) [51]	Halazun et al. (1974) [52]	Hau et al. (2019) [53]	Lipshultz et al. (1991) [3]	Pihkala et al. (1994) [49]	Velensek Prestor et al. (2000) [17]	Ram-meloo et al. (2000) [54]	Shim-momura et al. (2011) [55]	Steinherz et al. (1995) [48]	Turner-Gomes et al. (1996) [56]	Total
Author (date)												
Heart rate	147										0	147
Participants	216										19	235
Prevalence	68%										0%	63%
Tachycardia					8					1		9
Participants					96					2		98
Prevalence					8%					50%		9%
Bradycardia							4					4
Participants							46					46
Prevalence							9%					9%
Prolonged QT		0					2	0				2
Participants		23					46	90				159
Prevalence		0%					4%	0%				1%
ST segment			2			0	10		1			13
Participants			172			9	45		61			287
Prevalence			1%			0%	22%		2%			5%
T waves		7	11					1				19
Participants		23	172					90				285
Prevalence		30%	6%					1%				7%
Pathological Q waves		0	2									2
Participants		23	172									195
Prevalence		0%	1%									1%
No sinus rhythm		0						3				3
Participants		23						89				112
Prevalence		0%						3%				3%
Ventricular tachycardia					4		2	0				6
Participants					89		46	89				224
Prevalence					4%		4%	0%				3%
Ventricular ectopy					12					1		13
Participants					96					2		98
Prevalence					13%					50%		13%
Atrial ectopy					3							3
Participants					96							96
Prevalence					3%							3%
Arrhythmias				27								27
Participants				511								511
Prevalence				5%								5%
Ventricular arrhythmias										2		2
Participants										2		2
Prevalence										100%		100%
Ventricular premature contractions		3					3	3	2	1		12
Participants		23					44	89	59	2		217
Prevalence		13%					7%	4%	3%	50%		6%
Prolonged QRS interval								1				1
Participants								90				90
Prevalence								1%				1%
Premature atrial contractions								4				4
Participants								90				90
Prevalence								4%				4%
Anormal AV conduction		0										0
Participants		23										23
Prevalence		0%										0%
Right bundle branch block							1					1
Participants							46					46
Prevalence							2%					2%
Abnormal P waves			17									17
Participants			172									172
Prevalence			10%									10%
Second-degree AV block type I								3				3
Participants								89				89
Prevalence								3%				3%
Low QRS voltage			14			3		1				18
Participants			172			8		90				270
Prevalence			8%			38%		1%				7%
Abnormal QRS axis			9									9
Participants			172									172
Prevalence			5%									5%
Low T voltage			15									15
Participants			172									172
Prevalence			9%									9%
Abnormal T axis			11									11
Participants			172									172
Prevalence			6%									6%
Total	147	10	64	27	27	3	12	16	3	5	0	341
Participants	216	138	1204	511	377	17	228	806	120	8	19	3861
Prevalence	68%	7%	5%	5%	7%	18%	5%	2%	3%	63%	0%	9%

#### Heart rate abnormalities

Five studies reported heart rate abnormalities [3, 17, 48, 50, 56]. Two studies [3, 48] reported a prevalence of tachycardia of 9% (8–50%). One study [17] reported a prevalence of bradycardia of 9%. Two studies [50, 56] assessed whether the maximum heart rate during exercise was reached. The prevalence of not reaching maximum heart rate was 63% (0–68%).

#### Repolarization and depolarization disorders

For repolarization disorders, seven studies assessed QT and corrected QT (QTc) prolongation, and abnormal ST segments and T-wave changes. Three studies evaluated the QTc duration in a 12-lead ECG [17, 51, 54]. Two studies did not describe the precise method of evaluation [17, 51], the other study used Bazett’s formula [57] to calculate the QTc duration [54]. The prevalence of QTc prolongation defined as > 0.44 s (or > 0.46 s for females in Brouwer et al. [51]) was 1%. Three studies measured flattening T-waves [51, 52, 54]. The prevalence was 7% (1–30%). Four studies focused on ST segment abnormalities [17, 49, 52, 55]. The prevalence was 5% (0–22%). One study [3] reported a prevalence of 8% for abnormal ST segments and T-wave changes during exercise without further precision. One study [17] reported a prevalence of 7% for nonspecific ST segments and T-wave changes without further precision.

For depolarization disorders, two studies described their results [51, 52]. The prevalence of pathologic Q-waves in 12-lead ECG was 1% (0–1%).

#### Rhythm disturbances or abnormalities

Seven studies reported on different rhythm disturbances or abnormalities. Abnormal sinus rhythm was reported in two studies [51, 54]. Two studies [51, 54] reported a prevalence of 3% (0–3%) for abnormal sinus rhythm. The prevalence of arrhythmias (without categorization) was 5% and was reported in one study [53]. The prevalence of atrial ectopy was 3% and was reported in one study during exercise [3]. Ventricular arrhythmias were reported in five studies. One study [48] reported ventricular dysrhythmias without further precision in 100% of their participants (*N* = 2). Different ventricular arrhythmias were reported. The prevalence of ventricular premature contractions was 6% (3–50%) and was reported in five studies [17, 48, 51, 54, 55]. The prevalence of ventricular tachycardia was 3% (0–4%) and was reported in three studies [3, 17, 54]. The prevalence of ventricular ectopy was 13% (13–50%) and was reported in two studies [3, 48]. The prevalence of premature atrial contractions was 4% and was reported in one study [54]. The prevalence of prolonged QRS interval was 1% and was reported in one study [54].

#### Conduction disorders

Four studies reported conduction disorders [17, 51, 52, 54]. One study [51] reported that their participants presented no abnormal AV conduction disorders. A second-degree AV block type I was reported in 3% of participants in the other study [54]. One study reported a prevalence of 10% for atrial enlargement [52]. One study reported a prevalence of 2% for right bundle branch block [17].

#### Other

Three studies also reported cardiac electrical abnormalities that could not be assigned to one of the above subgroups [49, 52, 54]. A low QRS voltage was reported in 7% (1–38%) of patients. One study reported an incidence of abnormal T axis of 6% [52]. An abnormal QRS axis was also reported in 5% of patients. The incidence of low T voltage was 9% [52].

### Risk factors

Only three studies reported the association between cardiac electrical abnormalities and risk factors [17, 50, 54]. One study [50] performed linear regressions to explore the association between survivors’ maximal heart rate and outcomes of interest (i.e., cardiorespiratory fitness, total daily minutes of moderate to vigorous leisure physical activity, and prognostic risk groups). No significant differences were observed between males and females. No significant associations were reported between maximal heart rate and each outcome of interest (cardiorespiratory fitness, moderate to vigorous leisure physical activity, and prognostic risk groups). One study [54] did not present their results, but stated that they found no differences in any of the cardiac tests between boys and girls or between children below or above the age of four years at the time of treatment. One study [17] did not present their results, but stated that anthracycline dose had no influence on the incidence of cardiac abnormalities.

### Risk of bias

See Supplementary Tables S8, S9, and S10 for a complete assessment and description per study. In two studies, the definition used to describe an abnormal outcome was not provided [54, 56]. Also, two studies did not specify how the ECG was performed [48, 52]. In these cases, it was not clear whether the outcomes were measured in a valid and reliable way. In eight studies, confounding factors were not identified [17, 48–51, 53, 55, 56] and strategies to deal with confounding factors were not stated in seven of those studies [17, 48–51, 55, 56]. In three studies, important information with regard to patients’ characteristics was missing [48, 49, 52]. Overall, the risk of bias assessment shows several bias suggesting that included studies are at high risk of bias.

## Discussion

With the growing number of childhood ALL survivors every year, it seems essential to document and better understand the methods used to detect subclinical cardiac dysfunction in this unique population. This review shows that few studies (*N* = 11) have focused on the prevalence of cardiac electrical abnormalities in childhood ALL survivors. The ECG is a noninvasive and inexpensive tool that allows the detection of subclinical cardiac dysfunction in childhood ALL survivors. Included studies have found electrical heart problems after completion of treatment in this population, ranging from heart rate abnormalities to repolarization and depolarization disorders, rhythm disturbances or abnormalities, conduction disorders, and other disorders. This review advocates for improved cardiac monitoring with ECG and follow-up in childhood ALL survivors.

### Prevalence of cardiac electrical abnormalities

Overall, the prevalence of cardiac abnormalities in childhood ALL survivors is low after 4.4 to 23.0 years post-treatment. The included studies focused on cardiac electrical abnormalities to document childhood ALL survivors' cardiac status. Nevertheless, it is important to recognize that recently, published studies focused on cardiac electrical abnormalities to improve follow-up of survivorship in childhood cancer survivors by detecting subclinical cardiomyopathy [58]. These aims are, however, not specifically developed to study childhood ALL survivors' cardiac electrical abnormalities. Yet, childhood ALL survivors are a unique population group with a high risk of cardiac electrical remodeling due to chemotherapy exposition [59].

#### Heart rate abnormalities

Our review suggests that heart rate abnormalities occur frequently in childhood ALL survivors. One study reported tachycardia in one participant, but the authors did not specify if the event occurred during exercise or at rest [3]. Bradycardia occurred in a small number of childhood ALL survivors [17]. The discrepancy found in prevalence of abnormal maximal heart rate response to exercise may be due to differences in time since the end of the treatments, in the age of the participants at the time of evaluation, and in reliable criteria related to the verification of maximal exercise tests. Another reason that could explain the difference in prevalence is that the definition of abnormal heart rate response was not specified in these included studies [50, 56]. Only one included study verified that the exercise test was maximal with reliable criteria [50].

#### Repolarization and depolarization disorders

Prolongation of the QTc interval has been described as an early marker of clinical and subclinical cardiomyopathy. In childhood ALL survivors, there is no strong evidence of QTc prolongation after the end of treatment. In childhood cancer survivors, however, one study have reported a higher prevalence of QTc prolongation, compared to healthy controls [60]. Other studies have observed an association between QTc prolongation and subsequent left ventricular dysfunction in patients who exposed to anthracyclines [61–63].

Our review also reports a low prevalence of abnormal ST segments and T-wave changes. The low prevalence of abnormal ST segments and T-wave changes is also observed in childhood cancer survivor studies [17, 64, 65]. Abnormal ST segments and T-wave changes suggest that cancer treatment can cause cardiac remodeling in childhood ALL survivors. Hence, this would result from underlying processes, such as previous silent myocardial infarction or significant inflammation and fibrosis [66].

A low prevalence of pathologic Q-waves was reported in the studies that documented this parameter. In contrast, Mulrooney et al. [64] reported more major pathologic Q-waves and minor isolated Q/QS waves in childhood cancer survivors compared to the healthy control group. Pathologic Q waves may reflect cardiac remodeling and may indicate myocardial ischemia or the presence of chronic fibrosis in certain areas of the myocardium, or both. However, there is insufficient data to determine the clinical relevance of pathologic Q-waves in childhood ALL survivors.

Overall, these data suggest that childhood cancer survivors are more likely to develop chronic and pathologic QTc prolongation, ST segments and T-wave changes, and pathologic Q-waves. The prevalence may be over- or underestimated as only few of the included studies evaluated these abnormalities. Therefore, these anomalies deserve further study.

#### Rhythm disturbances or abnormalities 

The most studied cardiac electrical abnormalities parameter is rhythm disturbances. Overall, the prevalence was low, and it was lower than what has been previously found in childhood cancer survivors including ALL [62, 63, 67, 68]. Studies are scarce and the number of included patients is relatively small. For each specific abnormality, few studies have assessed their prevalence. This may over- or underestimate the true prevalence. As there might be a latency period for the development of rhythm disturbances or abnormalities, the length of follow-up in some studies may have been too short for participants to develop these problems. Also, assessment during or after exercise appears to increase the prevalence of some abnormalities (i.e., atrial ectopy, ventricular ectopy, and ventricular premature complexes).

The prevalence of ventricular premature contractions in the included studies is above what is expected in healthy individuals [69, 70]. Our review observed that childhood cancer survivors have an increased prevalence of arrhythmias and rhythm abnormalities compared with results from healthy children and young adults [65, 67]. An analysis by Markman et al. [62] showed that more clinically symptomatic arrhythmias were noted in patients who developed left ventricular dysfunction. This suggests that children receiving cancer treatment who are exposed to chemotherapy agents are more likely to develop chronic rhythm disturbances or abnormalities than those who have not been exposed.

#### Conduction disorders

Our review showed a low prevalence of conduction disorders despite that few studies have evaluated this parameter in childhood ALL survivors. In childhood cancer survivors, conduction disorders are also poorly studied. Studies have found a low to high prevalence of ventricular conduction disorders in childhood cancer survivors including ALL [17, 68]. Major atrioventricular conduction abnormalities are also more present in childhood cancer survivors than in healthy controls [64]. Conduction disorders following cancer treatment can be of varied clinical significance. Most often, degenerative conduction disorders are secondary to fibrosis of the tissues concerned.

#### Other

The prevalence of low QRS voltage was low in childhood ALL survivors. In one study, patients with low QRS voltage had additional evidence of cardiomyopathy, although still asymptomatic [22]. Decreases in QRS voltages have been associated with left ventricular dysfunction on echocardiogram after anthracycline usage [71, 72] and an increased risk of developing anthracycline-induced cardiomyopathy [61]. The prevalence of low QRS voltage found in our review is higher than in normal, thin subjects. Low QRS voltage may be associated with different situations, such as obesity, pericardial and pleural effusion, left ventricular hypertrophy, diffuse myocardial necrosis or fibrosis, emphysema, pulmonary infiltration, and hypothyroidism.

### Risk factors

Only three studies examined the associations between cardiac electrical abnormalities and possible risk factors in childhood ALL survivors. These studies did not find any associations [17, 50, 54]. In childhood cancer survivors, risk factors (e.g., radiotherapy involving the heart region, ≥ 300 mg/m^2^ of anthracyclines, male, and hypertension) for cardiac electrical abnormalities have been identified. The authors have suggested that this could help prognosis, risk stratification, and treatment of cardiomyopathies [58, 61, 62, 64, 68].

All studies included in this review studied anthracycline treatments. The actual received cumulative anthracycline dose was reported in the majority of studies and was very heterogenous, ranging from very low to very high doses. Only one study reported or discussed the drug effect of anthracyclines on electrical heart problems [17]. According to the authors, the number of survivors evaluated in their study was too small to draw conclusions on dose-dependence of the anthracycline effect. Studies also included different combinations of treatments without detailed information. No study reported the drug effect on any of the other treatments. However, several cancer treatments have been associated with arrhythmia [73, 74].

### Limitations

It is important to understand that the measurement methods and the quality of the included studies are very heterogeneous in childhood ALL survivors, as showed by our risk of bias assessment. This could limit the scope of our findings. As reported in this review, there is a high variability of study designs, inclusion and exclusion criteria, and patients’ characteristics, which makes inter-study comparisons challenging. Moreover, it is important to recognize that the scope of our review may have limited applicability to contemporary childhood ALL survivors. Nevertheless, the concerns raised in our review are timeless, especially those related to the cardiotoxicity and need to be further studied if researchers and clinicians want to provide better cardio-oncology follow-up care to their patients.

It would have been interesting to evaluate whether electrical cardiac abnormalities are more frequent in childhood ALL survivors than in the general population, but only three studies included healthy controls in their study design [3, 53, 55]. The quality of the included studies was reduced in part because items were often not reported or were not clearly defined (i.e., definition of an abnormal outcome, method of detection, confounding factors, patients’ characteristics, and treatment characteristics). In these cases, it was not clear whether outcomes were measured in a valid, reliable, and comparable way.

### Perspectives

This review has significant implication for research developments and patient care. Although the Children’s Oncology Group's guidelines advocate for the utilization of an ECG assessment as a baseline measure for all patients with a history of anthracycline exposure or thoracic radiation, it is important to note that the International Late Effects of Childhood Cancer Guideline Harmonization Group has reported certain gaps to use ECG. Our findings have the potential to address these gaps, particularly concerning the application of ECG alterations as prognostic indicators for future cardiomyopathy risk. Our comprehensive review has revealed a noteworthy prevalence of electrical cardiac abnormalities among childhood ALL survivors after completion of treatment. As discussed, childhood ALL survivors' cardiac function deteriorates after anthracycline administration and these survivors may become symptomatic over time. The assumption that electrical cardiac abnormalities reported in childhood ALL survivors could reflect an early stage of heart failure remains to be confirmed [44, 75]. Only one study included in this review evaluated cardiac electrical abnormalities’ clinical relevance and relation to cardiac dysfunction or future cardiac events [17]. The authors found no relationship between abnormal systolic or diastolic function and standard ECG results. In childhood cancer survivors, major ECG abnormalities were predictive of adverse outcomes, identifying a population that may warrant earlier and more comprehensive cardiac assessment and intervention. There is insufficient data to support the inclusion of cardiac electrical abnormalities endpoints in the surveillance of long-term childhood ALL survivors. Studies are necessary to better understand if these patients may benefit from increased screening frequency. Studies are also warranted to document if these patients may benefit from prophylactic therapy (i.e., beta-blockers, angiotensin-converting enzyme inhibitors) or other non-pharmacological interventions (i.e., exercise).

## Conclusion

This review reports that childhood ALL survivors have a low prevalence of cardiac electrical abnormalities several years after their treatment completion. Nevertheless, the lack of high-quality data precludes generalizing this to the entire population. Large prospective studies in childhood ALL survivors, clear definitions of cardiac electrical abnormalities, and comparison with a control group are warranted to better understand the presentation of cardiotoxicity, to improve clinical management, and to improve survival in childhood ALL survivors.

### Supplementary Information

**Additional file 1:** **Supplementary Table S1.** Search strategy for Pubmed. **Supplementary Table S2.** Search strategy for Ovid MEDLINE(R) and Epub Ahead of Print, In-Process, In-Data-Review & Other Non-Indexed Citations, Daily and Versions(R). **Supplementary Table S3.** Search strategy for Ovid All EBM Reviews. **Supplementary Table S4.** Search strategy for Ovid Embase. **Supplementary Table S5.** Search strategy for ISI Web of Science.

**Additional file 2:** **Supplementary Table S6.** Excluded studies. **Supplementary Table S7.** Studies with no data specific on childhood acute lymphoblastic leukemia survivors.

**Additional file 3:** **Supplementary Table S8.** Characteristics of included studies assessing prevalence and incidence of cardiac electrical remodeling. **Supplementary Table S9.** Prevalence of cardiac electrical remodeling. **Supplementary Table S10.** Prevalence of abnormalities.

**Additional file 4:** **Supplementary Table S11.** Risk of bias assessment for cross-sectional study. **Supplementary Table S12.** Risk of bias assessment for cohort study. **Supplementary Table S13.** Risk of bias assessment for randomized controlled trials. **Supplementary Table S14.** Risk of bias assessment for case control.

## Data Availability

The author confirms that all data generated or analysed during this study are included in this published article.

## Abbreviations

ALL: Acute lymphoblastic leukemia
AV: Atrioventricular
ECG: Electrocardiogram
QTc: Corrected QT interval
