# Supplementary material for: Cardiac electrical abnormalities in childhood acute lymphoblastic leukemia survivors: a systematic review
Source: Cardiooncology. 2023 Nov 11;9:40. doi: 10.1186/s40959-023-00188-9 (PMC10638753; doi:10.1186/s40959-023-00188-9)
Supplement: Supplementary file 1 — Additional file 1: Supplementary Table S1. Search strategy for Pubmed. Supplementary Table S2. Search strategy for Ovid MEDLINE(R) and Epub Ahead of Print, In-Process, In-Data-Review & Other Non-Indexed Citations, Daily and Versions(R). Supplementary Table S3. Search strategy for Ovid All EBM Reviews. Supplementary Table S4. Search strategy for Ovid Embase. Supplementary Table S5. Search strategy for ISI Web of Science. [file 40959_2023_188_MOESM1_ESM.docx]

## Supplementary Material. Search strategy for databases

## Supplementary Table S1: Search strategy for Pubmed

| #1 | Leukemia | Leukemia[mh] OR Hematologic Neoplasms[mh:noexp] OR Leukemi*[tiab] OR Leucocythaemi*[tiab] OR Leucocythemi*[tiab] OR "leucaemi*"[tiab] OR "leucemi*"[tiab] OR "leukaemi*"[tiab] OR Hematologic Neoplasm*[tiab] OR Haematologic Neoplasm*[tiab] OR Hematopoietic Neoplasm*[tiab] OR Haematopoietic Neoplasm*[tiab] OR Hematological Neoplasm*[tiab] OR Haematological Neoplasm*[tiab] OR Hematologic Cancer*[tiab] OR Haematologic Cancer*[tiab] OR Hematopoietic Cancer*[tiab] OR Haematopoietic Cancer*[tiab] OR Hematological Cancer*[tiab] OR Haematological Cancer*[tiab] OR Hematologic Malignanc*[tiab] OR Haematologic Malignanc*[tiab] OR Hematopoietic Malignanc*[tiab] OR Haematopoietic Malignanc*[tiab] OR Hematological Malignanc*[tiab] OR Haematological Malignanc*[tiab] OR Hematologic Tumor*[tiab] OR "Haematologic Tumor*"[tiab] OR Hematopoietic Tumor*[tiab] OR Haematopoietic Tumor*[tiab] OR Hematological Tumor*[tiab] OR Haematological Tumor*[tiab] OR "Hematologic Tumour*"[tiab] OR Haematologic Tumour*[tiab] OR Hematopoietic Tumour*[tiab] OR Haematopoietic Tumour*[tiab] OR "Hematological Tumour*"[tiab] OR Haematological Tumour*[tiab] OR blood cancer*[tiab] OR blood neoplasm*[tiab] OR blood tumor*[tiab] OR blood tumour*[tiab] OR blood malignan*[tiab] |
| --- | --- | --- |
| #2 | Pediatric | Infant[MH] OR Child[MH] OR Adolescent[MH] OR Intensive Care Units, Pediatric[MH] OR Hospitals, Pediatric[MH] OR Pediatrics[MH] OR Pediatricians[MH] OR Child, Hospitalized[MH] OR Adolescent, Hospitalized[MH] OR newborn*[tiab] OR new born*[tiab] OR babie*[tiab] OR baby*[tiab] OR infant*[tiab] OR infancy[tiab] OR toddler*[tiab] OR preschool*[tiab] OR pre school*[tiab] OR child*[tiab] OR kid[tiab] OR kid'[tiab] OR kids[tiab] OR kid's[tiab] OR boy[tiab] OR boy'[tiab] OR boys[tiab] OR boy's[tiab] OR girl[tiab] OR girl'[tiab] OR girls[tiab] OR girl's[tiab] OR schoolchild*[tiab] OR juvenil*[tiab] OR preadolescen*[tiab] OR youth*[tiab] OR adolescen*[tiab] OR teen*[tiab] OR puber*[tiab] OR high school*[tiab] OR highschool*[tiab] OR secondary school*[tiab] OR paediatric*[tiab] OR pediatric*[tiab] OR PICU*[tiab] OR neonat*[tiab] OR neo nat*[tiab] |
| #3 | Pediatric cancer | (Neoplasms[mh:noexp] AND child[mh]) OR Childhood cancer*[tiab] OR Pediatric cancer*[tiab] OR Paediatric cancer*[tiab] OR Childhood neoplasm*[tiab] OR Pediatric neoplasm*[tiab] OR Paediatric neoplasm*[tiab] OR Childhood malignanc*[tiab] OR Pediatric malignanc*[tiab] OR Paediatric malignanc*[tiab] OR Childhood tumor*[tiab] OR Pediatric tumor*[tiab] OR Paediatric tumor*[tiab] OR Childhood tumour*[tiab] OR Pediatric tumour*[tiab] OR Paediatric tumour*[tiab] |
| #4 | Heart | Cardiovascular Diseases[mh] OR Heart[mh] OR heart[tiab] OR cardia*[tiab] OR cardio*[tiab] OR arrythm*[tiab] OR arrhythm*[tiab] OR dysrhythm*[tiab] OR myocard*[tiab] OR Pericardi*[tiab] OR Ventric*[tiab] OR endocard*[tiab] OR tachycardi*[tiab] OR Tachyarr*[tiab] |
| #5 | Cardiac remodeling | Cardiomegaly[mh] OR Ventricular remodeling[mh] OR Remodel*[tiab] OR repolari*[tiab] OR ((interval[tiab] OR QT[tiab] OR QTc[tiab]) AND (prolong*[tiab] OR dispers*[tiab] OR shorten*[tiab] OR longer[tiab])) OR ((electrocardio*[tiab] OR echocardio*[tiab] OR electro-cardio*[tiab] OR echo-cardio*[tiab] OR ECG[tiab] OR ECGs[tiab] OR EKG[tiab]) AND (abnormal*[tiab] OR anormal*[tiab] OR anomal*[tiab])) OR wall thickness[tiab] OR Decompensat*[tiab] OR Hypertroph*[tiab] OR Dilation[tiab] OR Dilatation[tiab] OR cardiomegal*[tiab] OR enlarge*[tiab] OR expansion[tiab] |
| #6 | Combinaison | ((#1 AND #2) OR #3) AND #4 AND #5 AND (english[LA] OR French[LA] OR spanish[LA])  503 résultats |

## Supplementary Table S2: Search strategy for Ovid MEDLINE(R) and Epub Ahead of Print, In-Process, In-Data-Review & Other Non-Indexed Citations, Daily and Versions(R)

| 1 | Leukemia | Exp Leukemia/ OR Hematologic Neoplasms/ OR (Leukemi* OR Leucocythaemi* OR Leucocythemi* OR leucaemi* OR leucemi* OR leukaemi* OR Hematologic Neoplasm* OR Haematologic Neoplasm* OR Hematopoietic Neoplasm* OR Haematopoietic Neoplasm* OR Hematological Neoplasm* OR Haematological Neoplasm* OR Hematologic Cancer* OR Haematologic Cancer* OR Hematopoietic Cancer* OR Haematopoietic Cancer* OR Hematological Cancer* OR Haematological Cancer* OR Hematologic Malignanc* OR Haematologic Malignanc* OR Hematopoietic Malignanc* OR Haematopoietic Malignanc* OR Hematological Malignanc* OR Haematological Malignanc* OR Hematologic Tumor* OR Haematologic Tumor* OR Hematopoietic Tumor* OR Haematopoietic Tumor* OR Hematological Tumor* OR Haematological Tumor* OR Hematologic Tumour* OR Haematologic Tumour* OR Hematopoietic Tumour* OR Haematopoietic Tumour* OR Hematological Tumour* OR Haematological Tumour* OR blood cancer* OR blood neoplasm* OR blood tumor* OR blood tumour* OR blood malignan*).ti,ab,kw,kf |
| --- | --- | --- |
| 2 | Pediatric | Exp Infant/ OR exp Child/ OR Adolescent/ OR exp Intensive Care Units, Pediatric/ OR Hospitals, Pediatric/ OR Pediatrics/ OR Pediatricians/ OR Child, Hospitalized/ OR Adolescent, Hospitalized/ OR (newborn* OR new born* OR babie* OR baby* OR infant* OR infancy OR toddler* OR preschool* OR pre school* OR child* OR kid OR kid' OR kids OR kid's OR boy OR boy' OR boys OR boy's OR girl OR girl' OR girls OR girl's OR schoolchild* OR juvenil* OR preadolescen* OR youth* OR adolescen* OR teen* OR puber* OR high school* OR highschool* OR secondary school* OR paediatric* OR pediatric* OR PICU* OR neonat* OR neo nat*).ti,ab,kw,kf |
| 3 | Pediatric cancer | (Neoplasms/ AND child/) OR (Childhood cancer* OR Pediatric cancer* OR Paediatric cancer* OR Childhood neoplasm* OR Pediatric neoplasm* OR Paediatric neoplasm* OR Childhood malignanc* OR Pediatric malignanc* OR Paediatric malignanc* OR Childhood tumor* OR Pediatric tumor* OR Paediatric tumor* OR Childhood tumour* OR Pediatric tumour* OR Paediatric tumour*).ti,ab,kw,kf |
| 4 | Heart | Exp Cardiovascular Diseases/ OR exp Heart/ OR (heart OR cardia* OR cardio* OR arrythm* OR arrhythm* OR dysrhythm* OR myocard* OR Pericardi* OR Ventric* OR endocard* OR tachycardi* OR Tachyarr*).ti,ab,kw,kf |
| 5 | Cardiac remodeling | Exp Cardiomegaly/ OR exp Ventricular remodeling/ OR (Remodel* OR repolari* OR ((interval OR QT OR QTc) AND (prolong* OR dispers* OR shorten* OR longer)) OR ((electrocardio* OR echocardio* OR electro-cardio* OR echo-cardio* OR ECG OR ECGs OR EKG) AND (abnormal* OR anormal* OR anomal*)) OR wall thickness OR Decompensat* OR Hypertroph* OR Dilation OR Dilatation OR cardiomegal* OR enlarge* OR expansion).ti,ab,kw,kf |
| 6 | Combinaison | ((1 AND 2) OR 3) AND 4 AND 5 AND (english OR French OR spanish).lg  500 résultats |

## Supplementary Table S3: Search strategy for Ovid All EBM Reviews

| 1 | Leukemia | Exp Leukemia/ OR Hematologic Neoplasms/ OR (Leukemi* OR Leucocythaemi* OR Leucocythemi* OR leucaemi* OR leucemi* OR leukaemi* OR Hematologic Neoplasm* OR Haematologic Neoplasm* OR Hematopoietic Neoplasm* OR Haematopoietic Neoplasm* OR Hematological Neoplasm* OR Haematological Neoplasm* OR Hematologic Cancer* OR Haematologic Cancer* OR Hematopoietic Cancer* OR Haematopoietic Cancer* OR Hematological Cancer* OR Haematological Cancer* OR Hematologic Malignanc* OR Haematologic Malignanc* OR Hematopoietic Malignanc* OR Haematopoietic Malignanc* OR Hematological Malignanc* OR Haematological Malignanc* OR Hematologic Tumor* OR Haematologic Tumor* OR Hematopoietic Tumor* OR Haematopoietic Tumor* OR Hematological Tumor* OR Haematological Tumor* OR Hematologic Tumour* OR Haematologic Tumour* OR Hematopoietic Tumour* OR Haematopoietic Tumour* OR Hematological Tumour* OR Haematological Tumour* OR blood cancer* OR blood neoplasm* OR blood tumor* OR blood tumour* OR blood malignan*).ti,ab,kw,kf |
| --- | --- | --- |
| 2 | Pediatric | Exp Infant/ OR exp Child/ OR Adolescent/ OR exp Intensive Care Units, Pediatric/ OR Hospitals, Pediatric/ OR Pediatrics/ OR Pediatricians/ OR Child, Hospitalized/ OR Adolescent, Hospitalized/ OR (newborn* OR new born* OR babie* OR baby* OR infant* OR infancy OR toddler* OR preschool* OR pre school* OR child* OR kid OR kid' OR kids OR kid's OR boy OR boy' OR boys OR boy's OR girl OR girl' OR girls OR girl's OR schoolchild* OR juvenil* OR preadolescen* OR youth* OR adolescen* OR teen* OR puber* OR high school* OR highschool* OR secondary school* OR paediatric* OR pediatric* OR PICU* OR neonat* OR neo nat*).ti,ab,kw,kf |
| 3 | Pediatric cancer | (Neoplasms/ AND child/) OR (Childhood cancer* OR Pediatric cancer* OR Paediatric cancer* OR Childhood neoplasm* OR Pediatric neoplasm* OR Paediatric neoplasm* OR Childhood malignanc* OR Pediatric malignanc* OR Paediatric malignanc* OR Childhood tumor* OR Pediatric tumor* OR Paediatric tumor* OR Childhood tumour* OR Pediatric tumour* OR Paediatric tumour*).ti,ab,kw,kf |
| 4 | Heart | Exp Cardiovascular Diseases/ OR exp Heart/ OR (heart OR cardia* OR cardio* OR arrythm* OR arrhythm* OR dysrhythm* OR myocard* OR Pericardi* OR Ventric* OR endocard* OR tachycardi* OR Tachyarr*).ti,ab,kw,kf |
| 5 | Cardiac remodeling | Exp Cardiomegaly/ OR exp Ventricular remodeling/ OR (Remodel* OR repolari* OR ((interval OR QT OR QTc) AND (prolong* OR dispers* OR shorten* OR longer)) OR ((electrocardio* OR echocardio* OR electro-cardio* OR echo-cardio* OR ECG OR ECGs OR EKG) AND (abnormal* OR anormal* OR anomal*)) OR wall thickness OR Decompensat* OR Hypertroph* OR Dilation OR Dilatation OR cardiomegal* OR enlarge* OR expansion).ti,ab,kw,kf |
| 6 | Combinaison | ((1 AND 2) OR 3) AND 4 AND 5 AND (english OR French OR spanish).lg  44 résultats |

## Supplementary Table S4: Search strategy for Ovid Embase

| 1 | Leukemia | Exp Leukemia/ OR hematologic disease/ OR (Leukemi* OR Leucocythaemi* OR Leucocythemi* OR leucaemi* OR leucemi* OR leukaemi* OR Hematologic Neoplasm* OR Haematologic Neoplasm* OR Hematopoietic Neoplasm* OR Haematopoietic Neoplasm* OR Hematological Neoplasm* OR Haematological Neoplasm* OR Hematologic Cancer* OR Haematologic Cancer* OR Hematopoietic Cancer* OR Haematopoietic Cancer* OR Hematological Cancer* OR Haematological Cancer* OR Hematologic Malignanc* OR Haematologic Malignanc* OR Hematopoietic Malignanc* OR Haematopoietic Malignanc* OR Hematological Malignanc* OR Haematological Malignanc* OR Hematologic Tumor* OR Haematologic Tumor* OR Hematopoietic Tumor* OR Haematopoietic Tumor* OR Hematological Tumor* OR Haematological Tumor* OR Hematologic Tumour* OR Haematologic Tumour* OR Hematopoietic Tumour* OR Haematopoietic Tumour* OR Hematological Tumour* OR Haematological Tumour* OR blood cancer* OR blood neoplasm* OR blood tumor* OR blood tumour* OR blood malignan*).ti,ab,kw |
| --- | --- | --- |
| 2 | Pediatric | Exp juvenile/ OR exp pediatric intensive care unit/ OR Pediatrics/ OR Pediatricians/ OR (newborn* OR new born* OR babie* OR baby* OR infant* OR infancy OR toddler* OR preschool* OR pre school* OR child* OR kid OR kid' OR kids OR kid's OR boy OR boy' OR boys OR boy's OR girl OR girl' OR girls OR girl's OR schoolchild* OR juvenil* OR preadolescen* OR youth* OR adolescen* OR teen* OR puber* OR high school* OR highschool* OR secondary school* OR paediatric* OR pediatric* OR PICU* OR neonat* OR neo nat*).ti,ab,kw |
| 3 | Pediatric cancer | (Neoplasm/ AND exp child/) OR (Childhood cancer* OR Pediatric cancer* OR Paediatric cancer* OR Childhood neoplasm* OR Pediatric neoplasm* OR Paediatric neoplasm* OR Childhood malignanc* OR Pediatric malignanc* OR Paediatric malignanc* OR Childhood tumor* OR Pediatric tumor* OR Paediatric tumor* OR Childhood tumour* OR Pediatric tumour* OR Paediatric tumour*).ti,ab,kw |
| 4 | Heart | Exp Cardiovascular Disease/ OR exp Heart/ OR (heart OR cardia* OR cardio* OR arrythm* OR arrhythm* OR dysrhythm* OR myocard* OR Pericardi* OR Ventric* OR endocard* OR tachycardi* OR Tachyarr*).ti,ab,kw |
| 5 | Cardiac remodeling | Exp Cardiomegaly/ OR heart ventricle remodeling/ OR (Remodel* OR repolari* OR ((interval OR QT OR QTc) AND (prolong* OR dispers* OR shorten* OR longer)) OR ((electrocardio* OR echocardio* OR electro-cardio* OR echo-cardio* OR ECG OR ECGs OR EKG) AND (abnormal* OR anormal* OR anomal*)) OR wall thickness OR Decompensat* OR Hypertroph* OR Dilation OR Dilatation OR cardiomegal* OR enlarge* OR expansion).ti,ab,kw |
| 6 | Combinaison | ((1 AND 2) OR 3) AND 4 AND 5 AND (english OR French OR spanish).lg  1444 résultats |

## Supplementary Table S5: Search strategy for ISI Web of Science

| #1 | Leukemia | TS=(Leukemi* OR Leucocythaemi* OR Leucocythemi* OR leucaemi* OR leucemi* OR leukaemi* OR Hematologic Neoplasm* OR Haematologic Neoplasm* OR Hematopoietic Neoplasm* OR Haematopoietic Neoplasm* OR Hematological Neoplasm* OR Haematological Neoplasm* OR Hematologic Cancer* OR Haematologic Cancer* OR Hematopoietic Cancer* OR Haematopoietic Cancer* OR Hematological Cancer* OR Haematological Cancer* OR Hematologic Malignanc* OR Haematologic Malignanc* OR Hematopoietic Malignanc* OR Haematopoietic Malignanc* OR Hematological Malignanc* OR Haematological Malignanc* OR Hematologic Tumor* OR Haematologic Tumor* OR Hematopoietic Tumor* OR Haematopoietic Tumor* OR Hematological Tumor* OR Haematological Tumor* OR Hematologic Tumour* OR Haematologic Tumour* OR Hematopoietic Tumour* OR Haematopoietic Tumour* OR Hematological Tumour* OR Haematological Tumour* OR blood cancer* OR blood neoplasm* OR blood tumor* OR blood tumour* OR blood malignan*) |
| --- | --- | --- |
| #2 | Pediatric | TS=(newborn* OR new born* OR babie* OR baby* OR infant* OR infancy OR toddler* OR preschool* OR pre school* OR child* OR kid OR kid' OR kids OR kid's OR boy OR boy' OR boys OR boy's OR girl OR girl' OR girls OR girl's OR schoolchild* OR juvenil* OR preadolescen* OR youth* OR adolescen* OR teen* OR puber* OR high school* OR highschool* OR secondary school* OR paediatric* OR pediatric* OR PICU* OR neonat* OR neo nat*) |
| #3 | Pediatric cancer | TS=(Childhood cancer* OR Pediatric cancer* OR Paediatric cancer* OR Childhood neoplasm* OR Pediatric neoplasm* OR Paediatric neoplasm* OR Childhood malignanc* OR Pediatric malignanc* OR Paediatric malignanc* OR Childhood tumor* OR Pediatric tumor* OR Paediatric tumor* OR Childhood tumour* OR Pediatric tumour* OR Paediatric tumour*) |
| #4 | Heart | TS=(heart OR cardia* OR cardio* OR arrythm* OR arrhythm* OR dysrhythm* OR myocard* OR Pericardi* OR Ventric* OR endocard* OR tachycardi* OR Tachyarr*) |
| #5 | Cardiac remodeling | TS=(Remodel* OR repolari* OR ((interval OR QT OR QTc) AND (prolong* OR dispers* OR shorten* OR longer)) OR ((electrocardio* OR echocardio* OR electro-cardio* OR echo-cardio* OR ECG OR ECGs OR EKG) AND (abnormal* OR anormal* OR anomal*)) OR wall thickness OR Decompensat* OR Hypertroph* OR Dilation OR Dilatation OR cardiomegal* OR enlarge* OR expansion) |
| #6 | Combinaison | ((#1 AND #2) OR #3) AND #4 AND #5  Refined By:Languages: English or French or Spanish  1055 résultats |
