# Supplementary material for: Cardiac electrical abnormalities in childhood acute lymphoblastic leukemia survivors: a systematic review
Source: Cardiooncology. 2023 Nov 11;9:40. doi: 10.1186/s40959-023-00188-9 (PMC10638753; doi:10.1186/s40959-023-00188-9)
Supplement: Supplementary file 2 — Additional file 2: Supplementary Table S6. Excluded studies. Supplementary Table S7. Studies with no data specific on childhood acute lymphoblastic leukemia survivors. [file 40959_2023_188_MOESM2_ESM.docx]

**Supplementary Table S6: Excluded studies**

| **Authors, year** | **Reason** |
| --- | --- |
| Gulen et al., 2007 | No incidence or prevalence in the outcome |
| Long et al., 2020 | No incidence or prevalence in the outcome |
| Schwartz et al., 1993 | No incidence or prevalence in the outcome |
| Steinherz et al., 1991 | Overlapping cohort |

**Supplementary Table S7: Studies with no data specific on childhood acute lymphoblastic leukemia survivors**

| **Authors, year** | **Answer** |
| --- | --- |
| Allen, 2001 | Mailing errors |
| Behrouzian, 2020 | No answer |
| Benatar, 2015 | No answer |
| Bender, 1984 | Mailing errors* |
| Broberg, 2023 | No answer |
| Chow, 2022 | Data not available from the corresponding author |
| Cifra, 2018 | No answer |
| Cox, 2008 | Mailing errors* |
| Desai, 2019 | No answer |
| Gupta, 2002 | No answer |
| Gupta, 2009 | No answer |
| Gupta, 2021 | Data not available from the corresponding author |
| Hesseling, 1999 | Mailing errors* |
| Hogan, 2013 | Data not available from the corresponding author |
| Hongkan, 2009 | No answer |
| Hudson, 2007 | No answer |
| Jakacki, 1993 | No answer |
| Jakacki, 1993 (2) | No answer |
| Kesavapillai, 2019 | Mailing errors* |
| Kocabas, 2014 | No answer |
| Lang, 1995 | Mailing errors* |
| Larsen, 1992 | No answer |
| Lönnerholm, 1999 | Mailing errors* |
| Markman, 2017 | Mailing errors* |
| Mladosievicová, 1998 | No answer |
| Mladosievicova, 2000 | No answer |
| Mulrooney, 2016 | No answer |
| Mulrooney, 2020 | No answer |
| Mulrooney, 2017 | No answer |
| Pihkala, 1995 | Data not available from the corresponding author |
| Pourier, 2015 | No answer |
| Pourier, 2016 | No answer |
| Tamminga, 1992 | No answer |
| Tsuda, 2023 | No answer |
| Uchikoba, 2010 | No answer |
| Urbanova, 2010 | No answer |
| Velensek, 2008 | No answer |

*Mailling errors: available email address not working
