# Supplementary material for: Cardiac electrical abnormalities in childhood acute lymphoblastic leukemia survivors: a systematic review
Source: Cardiooncology. 2023 Nov 11;9:40. doi: 10.1186/s40959-023-00188-9 (PMC10638753; doi:10.1186/s40959-023-00188-9)
Supplement: Supplementary file 3 — Additional file 3: Supplementary Table S8. Characteristics of included studies assessing prevalence and incidence of cardiac electrical remodeling. Supplementary Table S9. Prevalence of cardiac electrical remodeling. Supplementary Table S10. Prevalence of abnormalities. [file 40959_2023_188_MOESM3_ESM.docx]

**Supplementary Table S8. Risk of bias assessment for cross-sectional study**

|  | **Q1** | **Q2** | **Q3** | **Q4** | **Q5** | **Q6** | **Q7** | **Q8** |
| --- | --- | --- | --- | --- | --- | --- | --- | --- |
| Bertrand et al., 2021 | YES | YES | YES | YES | UNCLEAR | UNCLEAR | YES | YES |
| Brouwer et al., 2007 | UNCLEAR | YES | YES | YES | NO | NO | YES | YES |
| Lipshultz et al., 1991 | NO | YES | YES | YES | YES | YES | YES | YES |
| Velensek Prestor et al., 2000 | YES | YES | YES | UNCLEAR | NO | NO | YES | YES |
| Shimomura et al., 2011 | YES | YES | YES | YES | NO | NO | NO | YES |
| Steinherz et al., 1995 | UNCLEAR | YES | YES | YES | NO | NO | NO | NO |
| Turner et al.,1996 | YES | YES | YES | YES | NO | NO | YES | YES |

|  | **Question** |
| --- | --- |
| **Q1** | Were the criteria for inclusion in the sample clearly defined? |
| **Q2** | Were the study subjects and the setting described in detail? |
| **Q3** | Was the exposure measured in a valid and reliable way? |
| **Q4** | Were objective, standard criteria used for measurement of the condition? |
| **Q5** | Were confounding factors identified? |
| **Q6** | Were strategies to deal with confounding factors stated? |
| **Q7** | Were the outcomes measured in a valid and reliable way? |
| **Q8** | Was appropriate statistical analysis used? |

**Supplementary Table S9: Risk of bias assessment for cohort study**

|  | **Q1** | **Q2** | **Q3** | **Q4** | **Q5** | **Q6** | **Q7** | **Q8** | **Q9** | **Q10** | **Q11** |
| --- | --- | --- | --- | --- | --- | --- | --- | --- | --- | --- | --- |
| Pihkala et al., 1994 | UNCLEAR | YES | YES | NO | NO | UNCLEAR | NO | YES | UNCLEAR | UNCLEAR | YES |
| Eva Hau et al., 2019 | YES | YES | YES | UNCLEAR | YES | UNCLEAR | YES | UNCLEAR | NO | NO | YES |

|  | **Question** |
| --- | --- |
| **Q1** | Were the two groups similar and recruited from the same population? |
| **Q2** | Were the exposures measured similarly to assign people to both exposed and unexposed groups? |
| **Q3** | Was the exposure measured in a valid and reliable way? |
| **Q4** | Were confounding factors identified? |
| **Q5** | Were strategies to deal with confounding factors stated? |
| **Q6** | Were the groups/participants free of the outcome at the start of the study (or at the moment of exposure)? |
| **Q7** | Were the outcomes measured in a valid and reliable way? |
| **Q8** | Was the follow up time reported and sufficient to be long enough for outcomes to occur? |
| **Q9** | Was follow up complete, and if not, were the reasons to loss to follow up described and explored? |
| **Q10** | Were strategies to address incomplete follow up utilized? |
| **Q11** | Was appropriate statistical analysis used? |

**Supplementary Table S10: Risk of bias assessment for randomized controlled trials**

|  | **Q1** | **Q2** | **Q3** | **Q4** | **Q5** | **Q6** | **Q7** | **Q8** | **Q9** | **Q10** | **Q11** | **Q12** | **Q13** |
| --- | --- | --- | --- | --- | --- | --- | --- | --- | --- | --- | --- | --- | --- |
| Rammeloo et al., 2011 | NO | NO | UNCLEAR | NO | NO | NO | YES | NO | UNCLEAR | YES | YES | UNCLEAR | UNCLEAR |
| Halazun et al., 1974 | UNCLEAR | NO | NO | NO | NO | NO | UNCLEAR | NO | UNCLEAR | UNCLEAR | UNCLEAR | NO | UNCLEAR |

|  | **Question** |
| --- | --- |
| **Q1** | Was true randomization used for assignment of participants to treatment groups? |
| **Q2** | Was allocation to treatment groups concealed? |
| **Q3** | Were treatment groups similar at the baseline? |
| **Q4** | Were participants blind to treatment assignment? |
| **Q5** | Were those delivering treatment blind to treatment assignment? |
| **Q6** | Were outcomes assessors blind to treatment assignment? |
| **Q7** | Were treatment groups treated identically other than the intervention of interest? |
| **Q8** | Was follow up complete and if not, were differences between groups in terms of their follow up adequately described and analyzed? |
| **Q9** | Were participants analyzed in the groups to which they were randomized? |
| **Q10** | Were outcomes measured in the same way for treatment groups? |
| **Q11** | Were outcomes measured in a reliable way? |
| **Q12** | Was appropriate statistical analysis used? |
| **Q13** | Was the trial design appropriate, and any deviations from the standard RCT design (individual randomization, parallel groups) accounted for in the conduct and analysis of the trial? |
