# Supplementary material for: Cardiac electrical abnormalities in childhood acute lymphoblastic leukemia survivors: a systematic review
Source: Cardiooncology. 2023 Nov 11;9:40. doi: 10.1186/s40959-023-00188-9 (PMC10638753; doi:10.1186/s40959-023-00188-9)
Supplement: Supplementary file 4 — Additional file 4: Supplementary Table S11. Risk of bias assessment for cross-sectional study. Supplementary Table S12. Risk of bias assessment for cohort study. Supplementary Table S13. Risk of bias assessment for randomized controlled trials. Supplementary Table S14. Risk of bias assessment for case control. [file 40959_2023_188_MOESM4_ESM.docx]

**Supplementary Table S11. Risk of bias assessment for cross-sectional study**

|  | **Q1** | **Q2** | **Q3** | **Q4** | **Q5** | **Q6** | **Q7** | **Q8** |
| --- | --- | --- | --- | --- | --- | --- | --- | --- |
| Bertrand et al., 2021 | YES | YES | YES | YES | UNCLEAR | UNCLEAR | YES | YES |
| Brouwer et al., 2007 | UNCLEAR | YES | YES | YES | NO | NO | YES | YES |
| Lipshultz et al., 1991 | NO | YES | YES | YES | YES | YES | YES | YES |
| Velensek Prestor et al., 2000 | YES | YES | YES | UNCLEAR | NO | NO | YES | YES |
| Shimomura et al., 2011 | YES | YES | YES | YES | NO | NO | NO | YES |
| Steinherz et al., 1995 | UNCLEAR | YES | YES | YES | NO | NO | NO | NO |
| Turner et al.,1996 | YES | YES | YES | YES | NO | NO | YES | YES |

|  | **Question** |
| --- | --- |
| **Q1** | Were the criteria for inclusion in the sample clearly defined? |
| **Q2** | Were the study subjects and the setting described in detail? |
| **Q3** | Was the exposure measured in a valid and reliable way? |
| **Q4** | Were objective, standard criteria used for measurement of the condition? |
| **Q5** | Were confounding factors identified? |
| **Q6** | Were strategies to deal with confounding factors stated? |
| **Q7** | Were the outcomes measured in a valid and reliable way? |
| **Q8** | Was appropriate statistical analysis used? |

**Supplementary Table S12: Risk of bias assessment for cohort study**

|  | **Q1** | **Q2** | **Q3** | **Q4** | **Q5** | **Q6** | **Q7** | **Q8** | **Q9** | **Q10** | **Q11** |
| --- | --- | --- | --- | --- | --- | --- | --- | --- | --- | --- | --- |
| Pihkala et al., 1994 | UNCLEAR | YES | YES | NO | NO | UNCLEAR | NO | YES | UNCLEAR | UNCLEAR | YES |

|  | **Question** |
| --- | --- |
| **Q1** | Were the two groups similar and recruited from the same population? |
| **Q2** | Were the exposures measured similarly to assign people to both exposed and unexposed groups? |
| **Q3** | Was the exposure measured in a valid and reliable way? |
| **Q4** | Were confounding factors identified? |
| **Q5** | Were strategies to deal with confounding factors stated? |
| **Q6** | Were the groups/participants free of the outcome at the start of the study (or at the moment of exposure)? |
| **Q7** | Were the outcomes measured in a valid and reliable way? |
| **Q8** | Was the follow up time reported and sufficient to be long enough for outcomes to occur? |
| **Q9** | Was follow up complete, and if not, were the reasons to loss to follow up described and explored? |
| **Q10** | Were strategies to address incomplete follow up utilized? |
| **Q11** | Was appropriate statistical analysis used? |

**Supplementary Table S13: Risk of bias assessment for randomized controlled trials**

|  | **Q1** | **Q2** | **Q3** | **Q4** | **Q5** | **Q6** | **Q7** | **Q8** | **Q9** | **Q10** | **Q11** | **Q12** | **Q13** |
| --- | --- | --- | --- | --- | --- | --- | --- | --- | --- | --- | --- | --- | --- |
| Rammeloo et al., 2011 | NO | NO | UNCLEAR | NO | NO | NO | YES | NO | UNCLEAR | YES | YES | UNCLEAR | UNCLEAR |
| Halazun et al., 1974 | UNCLEAR | NO | NO | NO | NO | NO | UNCLEAR | NO | UNCLEAR | UNCLEAR | UNCLEAR | NO | UNCLEAR |

|  | **Question** |
| --- | --- |
| **Q1** | Was true randomization used for assignment of participants to treatment groups? |
| **Q2** | Was allocation to treatment groups concealed? |
| **Q3** | Were treatment groups similar at the baseline? |
| **Q4** | Were participants blind to treatment assignment? |
| **Q5** | Were those delivering treatment blind to treatment assignment? |
| **Q6** | Were outcomes assessors blind to treatment assignment? |
| **Q7** | Were treatment groups treated identically other than the intervention of interest? |
| **Q8** | Was follow up complete and if not, were differences between groups in terms of their follow up adequately described and analyzed? |
| **Q9** | Were participants analyzed in the groups to which they were randomized? |
| **Q10** | Were outcomes measured in the same way for treatment groups? |
| **Q11** | Were outcomes measured in a reliable way? |
| **Q12** | Was appropriate statistical analysis used? |
| **Q13** | Was the trial design appropriate, and any deviations from the standard RCT design (individual randomization, parallel groups) accounted for in the conduct and analysis of the trial? |

**Supplementary Table S14: Risk of bias assessment for case control**

|  | **Q1** | **Q2** | **Q3** | **Q4** | **Q5** | **Q6** | **Q7** | **Q8** | **Q9** | **Q10** |
| --- | --- | --- | --- | --- | --- | --- | --- | --- | --- | --- |
| Eva Hau et al., 2019 | YES | YES | UNCLEAR | YES | YES | UNCLEAR | YES | YES | YES | YES |

|  | **Question** |
| --- | --- |
| **Q1** | Were the groups comparable other than the presence of disease in cases or the absence of disease in controls? |
| **Q2** | Were cases and controls matched appropriately? |
| **Q3** | Were the same criteria used for identification of cases and controls? |
| **Q4** | Was exposure measured in a standard, valid and reliable way? |
| **Q5** | Was exposure measured in the same way for cases and controls? |
| **Q6** | Were confounding factors identified? |
| **Q7** | Were strategies to deal with confounding factors stated? |
| **Q8** | Were outcomes assessed in a standard, valid and reliable way for cases and controls? |
| **Q9** | Was the exposure period of interest long enough to be meaningful? |
| **Q10** | Was appropriate statistical analysis used? |
